# Veno–veno–arterial extracorporeal membrane oxygenation treatment in patients with severe acute respiratory distress syndrome and septic shock

**DOI:** 10.1186/s13054-016-1205-9

**Published:** 2016-02-10

**Authors:** Hye Ju Yeo, Doosoo Jeon, Yun Seong Kim, Woo Hyun Cho, Dohyung Kim

**Affiliations:** 1 Department of Internal medicine, Pusan National University Yangsan Hospital, Medical Research Institute of Pusan National University, Geumo-ro 20, Beomeo-ri, Mulgeum-eup, Yangsan-si, Gyeongsangnam-do 626-770 Republic of Korea; 2 Department of Thoracic and Cardiovascular Surgery, Pusan National University Yangsan Hospital, the Research Institute for Convergence of Biomedical Science and Technology, Pusan National University Yangsan Hospital, Geumo-ro 20, Beomeo-ri, Mulgeum-eup, Yangsan-si, Gyeongsangnam-do 626-770 Republic of Korea

In acute respiratory distress syndrome (ARDS) with refractory septic shock, isolated veno–venous (VV) or veno–arterial (VA) extracorporeal membrane oxygenation (ECMO) may lead to differential hypoxia or inadequate tissue perfusion [1]. In this context, MacLaren et al. [2] showed that central ECMO improved the outcomes by guaranteeing systemic oxygenation without differential hypoxia. However, central ECMO has potential limitations due to its invasiveness and the lack of evidence in adult populations. Veno–veno–arterial (VVA) ECMO may offer effective oxygenation and hemodynamic support without differential hypoxia by regulating the return of oxygenated blood to the underperfused coronary and cerebral circulation [3–5]. Therefore, VVA mode can be an alternative treatment modality for ARDS patients with severe septic shock.

From October 2013 to March 2015, eight patients experienced septic shock with ARDS (seven men and one woman; average age 50.9 ± 5.9 years, range 18–71 years; five pneumonia-associated sepsis and three extra-pulmonary sepsis). The baseline patient characteristics are summarized in Additional file 1.

Before ECMO, the median mean arterial pressure (MAP) was 40 mmHg (interquartile range (IQR) 33–46), the median arterial lactate level was 7.8 mmol/L (IQR 6.3–16.3), and the median left ventricular ejection fraction was 42.5 % (IQR 23.5–50.0). Despite adequate fluid and vasopressor therapy, refractory shock proceeded. The median amount of fluid received was 4.7 l (IQR 4.3–4.9) and the median central venous oxygen saturation was 81.2 % (IQR 76.9–87.5). The median dose of norepinephrine was 0.7 μg/kg/min (IQR 0.6–0.8; also, vasopressin was used in all patients and six of the eight patients were also treated with epinephrine). All of the patients met the criteria for severe ARDS with a median PaO_2_/FiO_2_ of 57 (IQR 51.3–76.2; Table 1). The Institutional Review Board of Pusan National University Yangsan Hospital approved this study and waived the need for informed consent.

**Table 1:** Hemodynamics and arterial blood gas parameters before ECMO

Patient	Sex/Age	EF (%)	MAP (mmHg)	P/F ratio (mmHg)	PaCO2 (mmHg)	pH	Lactate (mmol/L)	Norepinephrine^a^	Vasopressin^a^	Fluid (L)	ScvO2 (%)
1	F/18	18	47	83.0	30	7.19	8.5	0.8	0.04	4.7	80.0
2	M/54	50	43	52.0	29	7.18	6.2	1.0	0.04	4.6	82.3
3	M/51	10	28	58.0	34	7.26	7.1	0.7	0.04	4.2	75.0
4	M/36	50	49	71.5	30	7.17	13.5	0.5	0.04	5.0	86.0
5	M/64	40	33	77.8	83	6.90	17.2	0.7	0.04	5.3	79.5
6	M/71	40	33	56.0	45	7.10	6.5	0.7	0.04	4.5	92.0
7	M/53	45	42	51.0	36	7.17	18.0	0.7	0.04	4.7	88.0
8	M/60	50	38	41.0	43	7.28	3.0	0.5	0.04	3.8	76.0

*ECMO* extracorporeal membrane oxygenation, *MAP* mean arterial pressure syndrome, *P/F ratio* PaO_2_/FiO_2_ ratio, *ScvO*_*2*_ central venous oxygen saturation (%)

^a^The dose is in μcg/kg/min

After VVA ECMO support, MAP increased, while the vasopressor dose and lactate level decreased and adequate oxygenation was sustained (Table 2). The median duration of vasopressor therapy was 24 h (IQR 18–72) and the median duration of VVA ECMO was 3.0 days (IQR 2.0–4.5). After 3 days, all patients had fully recovered from the refractory shock and they did not develop differential hypoxia. In addition, all patients were successfully weaned from arterial support and vasopressor. The overall survival rate was 50.0 %, and the successful weaning rate was 62.5 %. The number of patients is not enough to evaluate the feasibility, but VVA ECMO might be an alternative bridging strategy to assist the heart and lungs in patients with combined cardiopulmonary failure.

**Table 2:** Hemodynamic changes during VVA ECMO support

	Baseline	6 h	12 h	24 h	72 h
LVEF (%)	42.5 [23.5–50.0]			50.0 [40.0–50.0]	50.0 [40.0–55.0]
MAP (mmHg)	40.0	76.0	74.0	85.5	83.0
	[33.0–46.0]	[62.8–101.3]	[71.0–101.0]	[75.3–88.3]	[67.3–94.3]
Norepinephrine^a^	0.7 [0.6–0.8]	0.5 [0.2–0.6]	0.3 [0–0.6]	0.1 [0–0.1]	0 [0–0]
Epinephrine^a^	0.1 [0.0–0.2]	0 [0–0.1]	0 [0–0]	0 [0–0]	0 [0–0]
Arterial gas profile					
PaO_2_/FiO_2_	57.0	102.3	133.0	147.0	162.5
	[51.3–76.2]	[80.3–190.0]	[102.0–413.0]	[111.8–184.0]	[137.3–227.5]
Lactate (mmol/L)	7.8 [6.3–16.3]	5.5 [2.5–14.8]	6.3 [2.0–15.5]	7.0 [3.0–14.0]	5.0 [2.0–5.0]
pH	7.2 [7.1–7.2]	7.4 [7.3–7.5]	7.4 [7.3–7.4]	7.4 [7.4–7.5]	7.5 [7.4–7.5]

The data are presented as median [interquartile range]

*VVA* veno-venoarterial, *ECMO* extracorporeal membrane oxygenation, *LVEF* left ventricle ejection fraction, *MAP* mean arterial pressure

^a^The dose is in μcg/kg/min

## Additional file

Additional file 1:
**Baseline patient characteristics.** *F* female, *M* male. (DOCX 15 kb)

## Abbreviations

ARDS: Acute respiratory distress syndrome
ECMO: Extracorporeal membrane oxygenation
IQR: Interquartile range
MAP: Mean arterial pressure
VA: Veno–arterial
VV: Veno–venous
VVA: Veno–veno–arterial
