# Supplementary material for: Veno–veno–arterial extracorporeal membrane oxygenation treatment in patients with severe acute respiratory distress syndrome and septic shock
Source: Crit Care. 2016 Feb 10;20:28. doi: 10.1186/s13054-016-1205-9 (PMC4748570; doi:10.1186/s13054-016-1205-9)
Supplement: Additional file 1: — Baseline patient characteristics. F female, M male. (DOCX 15 kb) [file 13054_2016_1205_MOESM1_ESM.docx]

Electronic supplement 1. Baseline patient characteristics

| Patient | Sex/Age | Cause of ARDS | APACHE II | SOFA | Lung injury score |
| --- | --- | --- | --- | --- | --- |
| 1 | F/18 | Pneumonia, septic shock | 8 | 10 | 3.0 |
| 2 | M/54 | Extra-pulmonary septic shock | 25 | 16 | 3.25 |
| 3 | M/51 | Pneumonia, septic shock | 11 | 14 | 3.5 |
| 4 | M/36 | Extra-pulmonary septic shock | 20 | 18 | 3.25 |
| 5 | M/64 | Pneumonia, septic shock | 23 | 15 | 3.25 |
| 6 | M/71 | Pneumonia, septic shock | 13 | 12 | 3.5 |
| 7 | M/53 | Extra-pulmonary septic shock | 20 | 12 | 3.5 |
| 8 | M/60 | Pneumonia, septic shock | 20 | 12 | 3.5 |

ARDS, acute respiratory distress syndrome; APACHE II, Acute Physiology and Chronic Health Evaluation; SOFA, Sequential Organ Failure Assessment
